# Modeling Diet-Induced NAFLD and NASH in Rats: A Comprehensive Review

**DOI:** 10.3390/biomedicines9040378

**Published:** 2021-04-02

**Authors:** Lydie Carreres, Zuzana Macek Jílková, Guillaume Vial, Patrice N. Marche, Thomas Decaens, Hervé Lerat

**Affiliations:** 1 Institute for Advanced Biosciences, Research Center Inserm U 1209/CNRS 5309, 38700 La Tronche, France; lydie.carreres@univ-grenoble-alpes.fr (L.C.); zmacekjilkova@chu-grenoble.fr (Z.M.J.); patrice.marche@univ-grenoble-alpes.fr (P.N.M.); tdecaens@chu-grenoble.fr (T.D.); 2 Université Grenoble-Alpes, 38000 Grenoble, France; guillaume.vial@inserm.fr; 3 Inserm U 1300, Hypoxia PathoPhysiology (HP2), 38000 Grenoble, France; 4 Service D’hépato-Gastroentérologie, Pôle Digidune, CHU Grenoble Alpes, 38700 La Tronche, France; 5 Unité Mixte de Service UGA hTAG, Inserm US 046, CNRS UAR 2019, 38700 La Tronche, France

**Keywords:** NAFLD, NASH, fibrosis, metabolic syndrome, obesity, rats, diet

## Abstract

Nonalcoholic fatty liver disease (NAFLD) is the most common chronic liver disease, characterized by hepatic steatosis without any alcohol abuse. As the prevalence of NAFLD is rapidly increasing worldwide, important research activity is being dedicated to deciphering the underlying molecular mechanisms in order to define new therapeutic targets. To investigate these pathways and validate preclinical study, reliable, simple and reproducible tools are needed. For that purpose, animal models, more precisely, diet-induced NAFLD and nonalcoholic steatohepatitis (NASH) models, were developed to mimic the human disease. In this review, we focus on rat models, especially in the current investigation of the establishment of the dietary model of NAFLD and NASH in this species, compiling the different dietary compositions and their impact on histological outcomes and metabolic injuries, as well as external factors influencing the course of liver pathogenesis.

## 1. Introduction

### 1.1. Definition and Etiology of NAFLD and NASH

Nonalcoholic fatty liver disease (NAFLD) is the most common human chronic liver disease (CLD). It is characterized by hepatic triglyceride content of over 5% and the histological feature of simple steatosis, with no evidence of alcohol abuse [1]. It is the hepatic manifestation of metabolic syndrome [2], which is defined by the presence of at least three out of the five following features: obesity, hypertriglyceridemia, hyperglycemia, hypertension and low-level HDL cholesterol. NAFLD is a wide-spectrum disease, ranging from simple steatosis to nonalcoholic steatohepatitis (NASH), which may further progress to cirrhosis and end-stage liver disease hepatocellular carcinoma (HCC) [3].

Incidence of obesity is rapidly growing worldwide and is associated with an increased risk of NAFLD. In developed countries, approximately 30% of adults develop NAFLD [4], and between 20% to 30% of these individuals will subsequently develop NASH [5]. In overweight and diabetic individuals, NAFLD occurrence may rise to 50% and 80%, respectively [2].

**Figure 1:**
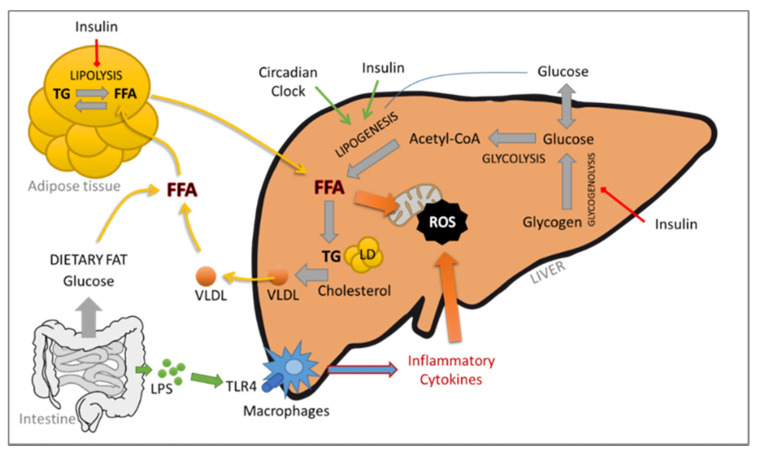
Schematic representation of the molecular dynamism in the “multiple parallel hits hypothesis”.

Moreover, it is considered that 20% of NAFLD patients will progress to NASH [4]. The risk of NASH increases with age; the male sex; and components of the metabolic syndrome, including diabetes, hypertension, obesity and dyslipidemia. NASH is characterized by the presence of steatosis, lobular inflammation, hepatocellular ballooning and liver injuries resulting in necroinflammation accompanied by fibrosis. The pathophysiology of NASH is not completely known. Initially, Day and James in 1998 proposed the “two-hit hypothesis”, in which the first hit was steatosis, which led to the second hit, oxidative stress, mainly due to the increase in lipotoxicity induced by the first step [6]. Due to the molecular dynamism and the multitude of factors, the “multiple parallel hits hypothesis” was proposed more recently [7]. It includes several factors that happen in parallel, such as diet, lifestyle, gut microbiota modification, epigenetic and genetic variations, mitochondrial dysfunctions, oxidative and/or endoplasmic reticulum (ER) stress, lipid metabolism deregulation and immune system modulations. Briefly, accumulation of lipids leading to a fatty liver can be mediated by several mechanisms, such as an increase in free fatty acids (FFAs) from an exacerbated lipolysis or intake of dietary fat, a decrease in FFA oxidation, an increase in de novo hepatic lipogenesis and a decrease in hepatic very low density lipoprotein (VLDL) triglyceride secretion. Hepatic accumulation of FFA and cholesterol leads to mitochondrial dysfunction and ER stress, resulting in tumor necrosis factor-α (TNF-α)-mediated liver damage and reactive oxygen species (ROS) formation. ER stress can interfere with various inflammatory pathways that are involved in the regulation of obesity-related insulin resistance and inflammation, such as the pathways of ROS, nuclear factor κB (NF-κB) and c-Jun N-terminal Kinase (JNK). The gut–liver axis is also affected by dietary intake, resulting in dysbiosis of the microbiota and production of inflammatory cytokines. Several toll-like receptors (TLR) expressed in the gut epithelium might respond to the nutritional lipids and affect systemic inflammation and insulin resistance. The contribution of the adipose tissue–liver axis is also critical. In fact, the adipose tissue is an active endocrine and immune organ capable of producing several mediators responsible for the crosstalk between the liver and the adipose tissue, such as leptin, IL-6 and TNF-α, which play a central role in NAFLD/NASH (see Figure 1).

NASH, obesity and type 2 diabetes mellitus are nowadays recognized as risk factors for the development of HCC [8]. NASH-related HCC is currently the most rapid-growing indication for liver transplant in HCC patients [9]. HCC is the most common form of liver cancer worldwide; it holds the sixth position in terms of cancer incidence and is the third-leading cause of cancer-related deaths [10]. Although the molecular pathogenesis of the hepatitis B and C viruses (HBV and HCV, respectively) and its contribution to HCC has been widely studied, that of NASH remains a challenge. The mechanisms leading to cancer development are not fully elucidated, but interestingly, cirrhosis might not be a prerequisite (for recent reviews, see [11,12,13]), suggesting the involvement of different or additional pathways.

**Figure 2:**
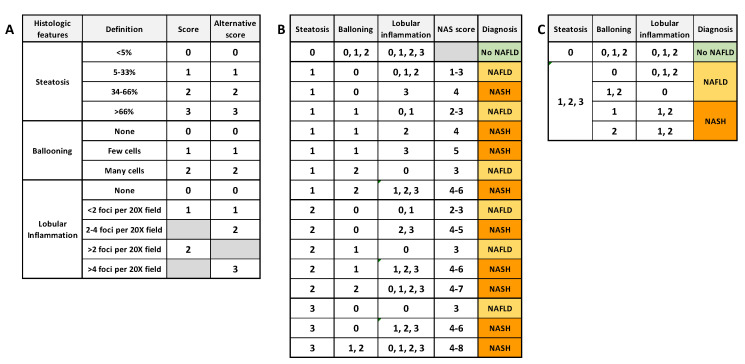
Common algorithms for NAFLD and NASH scoring. (**A**) semiquantitative histology scoring for each histologic feature. (**B**) Diagnosis algorithm based on NAS score (NASH if NAS score ≥ 4) [14]. (**C**) Diagnosis algorithm based on unweighted histologic features (NASH if all features present, whatever the score) [15].

Liver biopsies remain the gold standard to distinguish NAFLD from NASH and to diagnose NASH and HCC. This is an invasive and costly procedure in which clinicians have to manage the accuracy, pain and patient safety. Nevertheless, biopsies allow characterizing and evaluating in situ the grade and stage of NASH, which is not achievable using noninvasive tests. The NASH Clinical Research Network (CRN) proposed an “NAFLD activity score” (NAS) to stage NAFLD biopsies. This score is based on the unweighted sum of the score for steatosis (0–3), hepatocellular ballooning (0–2) and lobular inflammation (0–3) [14], or (0–2) [15]. Kleiner et al. [14] proposed that a NAS score ≤ 3 is considered as “no NASH” (NAFLD if steatosis ≥ 1) and a score ≥ 4 is considered as definitely afflicted with NASH. Bedosa et al. proposed steatosis score as criteria of entry and the presence of all histologic features to diagnose NASH. Figure 2 summarizes the algorithms commonly used for NASH and NAFLD histological determination. More recently, another NASH grading, the “steatosis–activity–fibrosis” (SAF) system was proposed. It is based on the semiquantitative scoring of steatosis (S), activity (A) and fibrosis (F), with steatosis being less weighted [15]. In this system, all features are needed to diagnose NASH, even with the lowest score. Ballooning degeneration of hepatocytes with or without fibrosis is the key feature that differentiates NASH from NAFLD.

### 1.2. In Vivo Modeling of Human NAFLD/NASH

Although the initiation and development of NAFLD are highly correlated with the markers for metabolic syndrome, all steps ranging from NAFLD to HCC and the mechanism underlying these transitions are under high scrutiny by researchers. However, they are still poorly understood due to the lack of relevant models and poorly transposable results. Since NAFLD is a systemic disease, its in vitro modeling using simple 2D cell cultures would be of limited interest. Research on liver organoids has made tremendous progress and proposes well-defined protocols that are useful for basic research on liver development and regeneration. Nonetheless, this approach bears serious limitations, such as limited access to human tissues and failure to recapitulate the multiple cell types of the liver (for review, see [16]). Several animal models have been proposed to mimic the pathophysiology and clinical features of human NAFLD and NASH. An adequate animal model should recapitulate the etiological, pathological, histological, proteomic and transcriptomic features of the human disease. It should develop from the same risk factors, such as metabolic syndrome (obesity, insulin resistance, dyslipidemia, type 2 diabetes mellitus) and display the histologic phenotype of steatosis, lobular inflammation, hepatocellular ballooning and fibrosis. It should be able to progress to advanced fibrosis, cirrhosis and ultimately HCC and show clear evidence of systemic inflammation. Furthermore, such a model should be reproducible, reliable, simple, easy and affordable for development and preclinical validation of new therapeutic targets. There are several ways to induce NAFLD and NASH in rodents, including genetic approaches, chemical treatments, special diets or even combinations of these. A few NASH models based on genetic modifications have been described: the Zucker fatty rats (fa/fa), which bear spontaneous mutations for leptin receptor; the stroke-prone spontaneously hypertensive 5/Dmcr rats (SHRSP5/Dmcr); the Otsuka Long–Evans Tokushima fatty rats (OLETF) [17]; the Cx32 dominant-negative transgenic rats (Cx32ΔTg) [18]; and the Leptin mutant rats (Lep^DI14/DI14^) [19]. These models usually displayed only a few features of NASH and required combination with special diets to worsen the phenotype [20,21], with sometimes limited success [22].

Diet-induced obesity (DIO) being the most common risk factor for NAFLD in humans, the animal model should mimic the origin of the human disease as closely as possible and follow the human dietary composition in order to generate highly transposable results. In this review, we focus on diet-induced models.

### 1.3. Rat versus Mouse in Liver Disease Modeling

Mice have been the animal of choice to model human diseases, including CLD, mainly because of the wide variety of mouse molecular tools available. However, homologous recombination, the full-genome sequence database and annotation for rats and CRISPR/Cas9 technology have contributed to the fast development of rat-dedicated tools and a genetically modified model, allowing researchers to model human disease in this rodent [23]. In their genome-scale network reconstruction, Blais et al. demonstrated that rats and humans share an overwhelming majority of their biochemical capabilities at the genome level, underscoring the important role of rats as a model organism for understanding human biology and disease [24]. In a study by Fuchs et al. [25], epidermal growth factor (EGF) expression was described in cirrhotic patients as a prognosis factor for survival, along with 186 other genes. This human genetic signature has been compared to rodent models of NASH-HCC, and only rats displayed a significantly equivalent signature. In contrast to mice, in a model of DIO, rats showed no significant intraindividual, intralobular or interlobular sampling variation for histological features or for liver biochemical read-outs [26]. In addition, rat models are thought to be more susceptible to high-fat diets (HFDs) and thus may display more severe and/or earlier histological features of NAFLD compared to mice [27]. Furthermore, preclinical NASH studies in mice are often challenged by the difficulties in obtaining a severe fibrotic response in most diet-induced models [28].

Other benefits of using rats include the quantities of samples available (e.g., blood volume, liver size), the capacity to train and the ease of surgical procedures. Although space and cost constraints are non-negligible, candidate drugs are routinely tested in rats (mainly in Sprague Dawley) to assess safety and efficacy before human clinical trials.

In this review, we focus on rat models, especially on the current investigation in the establishment of the dietary model of NAFLD and NASH in this species, compiling the different dietary compositions and their impact on histological outcomes and metabolic injuries, as well as external factors influencing the course of liver pathogenesis. When relevant, a short comparison with mice models will be presented.

## 2. Diet-Induced NAFLD and NASH Models in Rats

### 2.1. Amino Acid Modified Diets

#### 2.1.1. Methionine- and Choline-Deficient Diet

Methionine and choline amino acids are methyl-group donors that are essential for lipid metabolism. They are involved in hepatic β-oxidation and VLDL production. In some particular situations (namely malnutrition and alcohol abuse), when choline is lacking in the diet, methionine can be used to synthesize choline. A methionine- and choline-deficient diet (MCDD) is depleted in both of these essential amino acids and induces dysregulation of phospholipid synthesis, lipoprotein secretion and oxidative and ER stress [29]. The energy density of this diet is around 4300 kcal/kg. Thus, such diets are simple and effective ways to induce NAFLD in animals.

Sprague Dawley rats fed using the MCDD for 6–8 weeks developed severe steatosis, but low inflammation and no ballooning or fibrosis [30,31]. The observed NAS score was usually 4 [32]. Similar histological observations were reported in Wistar rats that were fed the MCDD for 13 weeks [33]. MCDD has the advantage of inducing strong steatosis in short-term experiments. In contrast, one of the weaknesses of this diet is the absence of obesity and metabolic syndrome. Animals showed lower serum levels of glucose, insulin triglyceride, leptin and cholesterol. Moreover, animals lost up to 40% of their weight after 10 weeks of MCDD feeding [34], mainly because of the reduction in corporal fat, and a proportional decrease in liver size, associated with lower calorie intake [35]. To counterbalance this effect, fat can be added to MCDD. In this case, animals developed metabolic syndrome, with an increase in body weight, adipose tissue weight, blood triglyceride levels and Homeostatic Model Assessment of Insulin Resistance (HOMA-IR) [36]. Between the Sprague Dawley, Long–Evans and Wistar rat models, the last-mentioned was more prone to developing steatosis and did not show liver weight loss [37].

Baicalein, a flavonoid contained in dried roots that shows promise as a chemopreventive compound, was tested in the MCCD model in order to prevent NASH development. Decreased triglyceride and cholesterol levels in the liver were noticed, along with a reduction in NAS histological score, apoptosis, inflammation and oxidative stress [26].

MCDD is widely used in mice as it induces a more severe form of NASH, with steatosis, inflammation, ballooning and fibrosis in short-time diets [38]. In a study comparing C57BL/6 mice to Wistar rats fed MCDD, the latter developed more steatosis, but at a slower rate than mice. In addition, necroinflammation was abundant in mice and very mild in rats [37]. This diet seemed to be more efficient in mice, explaining the large amount of data generated in MCDD mouse models compared to MCDD rat models. Nonetheless, rat and mouse MCDD models displayed weight loss and low levels of glucose, insulin and triglycerides in serum, which are all important hallmarks that define NASH.

#### 2.1.2. Choline-Deficient L-Amino Acid Defined Diet

The choline-deficient L-amino acid defined (CDAA) diet is similar to the MCDD, with a low quantity of methionine (0.1%) and an average caloric density of 4320 kcal/kg. This diet, administered to Fisher 344 or Wistar rats for 6–12 weeks, induced severe steatosis with inflammation but lacked ballooning phenotypes [39,40]. Animals were not overweight compared to the control group and did not develop metabolic syndrome. However, the CDAA diet offered a considerable advantage over the MCDD, since the animals subjected to the former developed severe liver fibrosis [41,42]. Antifibrotic therapeutic approaches have been tested using this model. Obeticholic acid, a farnesoid X receptor agonist, combined with a dipeptidyl peptidase-4 inhibitor (sitagliptin) reduced fibrotic gene expression, NAS and fibrosis histological score and levels of IL-6 and interferon gamma (IFN-γ) in the liver and intestine [42]. Impaired respiratory function of mitochondria was described in Wistar rats that were fed the CDDA diet for 16 weeks [43], and complex I dysfunction associated with increased H_2_O_2_ production [44] was observed after only a week on the CDAA diet.

A long period of the CDAA diet (for up to 40 weeks) in Wistar rats induced steatosis, severe fibrosis and, more interestingly, adenoma that progressed to HCC in 50% of the cases [45,46]. Similar results were reported by Kitade et al. on fa/+ control Zucker rats (displaying no leptin modification) that were fed the CDAA diet for 80 weeks [45,46]. When telmisartan, an angiotensin II type 1 receptor blocker, was given to CDAA-fed animals, hepatic fibrosis and preneoplastic lesions were inhibited and development of HCC was prevented [46].

Thus, according to the literature, the MCDD model mimics the histopathological features of NAFLD. However, in addition to the absence of metabolic syndrome in these models, such amino acid deficiency is not common in human diets, limiting their relevance when the etiology of NAFLD is considered.

Similar to the MCDD, the CDAA diet is not representative of the etiology of human NASH. Moreover, the typical features of NAFLD, namely metabolic syndrome, ballooning and obesity, were absent in this model. However, the report of HCC development upon long-term feeding of the CDAA diet is of high interest for NASH-to-HCC progression studies.

### 2.2. “Western-Style” Diets

A Western-style diet is characterized by excess refined carbohydrates, highly saturated ω-6 and trans fatty acids, low levels of ω-3 fatty acids and other long-chain polyunsaturated fatty acids (PUFAs) and lower levels of antioxidant vitamins and flavonoids. It has been shown to be proinflammatory and may cause metabolic syndrome in humans.

#### 2.2.1. High-Fat Diet

High-fat diet (HFD)-fed animals have been developed to reproduce this Western style of detrimental eating habits and to mimic the etiology of NAFLD/NASH in humans. The main characteristic of this type of diet is that energy intake comes from approximately 60% of fat (ranging from 45% to 75%) compared to less than 20% in a normal rodent diet.

A well-studied HFD is the Lieber–DeCarli liquid HFD containing 1000 kcal/L, of which 71% is fat-derived (mainly corn oil), 18% is derived from proteins and 11% is derived from carbohydrates. Sprague Dawley rats fed with this diet developed hyperglycemia, macrovesicular steatosis and mild inflammation, which is associated with an increase in hepatic TNF-α at 3 weeks [47]. Nevertheless, rats did not develop obesity when compared to the control group. Other researchers continued this diet for 6 weeks, but despite the longer HFD feeding, no ballooning or inflammation was seen [48,49,50]. Interestingly, increased ROS production was observed in the study by Vial et al. when Wistar rats were fed with 54% lipids (50% lard, 4% soya-bean oil *w*/*w*) for 8 weeks. ROS production was associated with inhibition of fatty acid oxidation and mitochondrial oxidative phosphorylation, suggesting a key role of mitochondria in HFD-induced NAFLD [51].

In a recent study from Emamat et al. [52], Sprague Dawley rats that were fed HFD (60% of total calories from butter-derived fat) for 7 weeks developed metabolic syndrome, encompassing the development of obesity, hypertriglyceridemia, hyperinsulinemia and hyperglycemia, which are associated with the development of NASH. Su et al. published similar results with rats that were fed the HFD (mainly derived from lard, with a total of 5210 kcal/kg) for 14 weeks and observed a NAS score of over 5 [53]. In another study, the HFD (5600 kcal/kg; saturated lipids 30.4 g/100 g, unsaturated lipids 5.3 g/100 g, originating from corn starch and coconut oil) was extended to 24 weeks [54], but with no additional worsening phenotypes. In contrast to these observations, the same diet (mainly derived from lard, with a total of 5210 kcal/kg) used in Wistar rats for 12 weeks did not induce NASH [55].

This clearly demonstrated that not all rodent models respond in the same manner to HFD due to genetic differences. Comparisons of studies are difficult due to the discrepancies in HFD composition, mainly in fat sources. The importance of fat sources in diet composition has been reported by Buettner et al. in a study using Wistar rats fed for 12 weeks with 42% of energy originating from lard, olive oil, coconut oil or fish oil fat. The most pronounced obesity and insulin resistance were observed with lard and olive oil fat, associated with liver steatosis and sterol response element-binding protein-1c (SREBP-1c)-dependent gene transcription upregulation.

In mice, HFD (60% fat) has been shown to induce strong steatosis with hyperlipidemia and hyperinsulinemia after 10 to 12 weeks of diet [56], while in rats subjected to the same diet, only 7 weeks were needed to observed these features [52]. Long-term HFD feeding in mice was not sufficient to promote inflammation or ballooning, while such a diet induced weak inflammation and ballooning in rats [57]. Thus, rats seemed more susceptible to HFD. Nevertheless, it has been reported that within experimental batches, a proportion of rats might not gain weight during HFD feeding. Consequently, some researchers usually choose to eliminate these animals and keep for their analyses only those animals that responded correctly to this parameter. Other researchers analyzed responders or nonresponders in separate groups (for example, [58]). However, it has been shown that rats that did not necessarily gain a lot of weight still have significant steatosis. In fact, Gauthier et al. [59] demonstrated that the development of NASH was linked more to dietary fat ingestion than body weight gain. The accumulation of ectopic fat in these obesity-resistant animals seemed to be higher, which might be more deleterious, and thus, such groups of rats should be analyzed with great interest.

To summarize, HFD rat models can replicate the metabolic parameters observed in NASH patients. While steatosis was highly induced by HFD, the histopathological outcomes of NASH, such as inflammation, ballooning and fibrosis, were mild or even absent. Moreover, HFD alone has never been described as inducing the development of HCC.

#### 2.2.2. Complemented High-Fat Diet

##### Atherogenic Diets (High-Fat and High-Cholesterol (HFD+C) Diet)

The main organ of cholesterol metabolism is the liver, and its homeostasis is dependent on its intake (endogenous and exogenous through dietary uptake), transport and catabolism. In studies on diet-induced NASH animal models, authors commonly added cholesterol in their procedures. The quantity can vary from 0.5% to 10%. Ichimura et al. demonstrated the role of cholesterol in this complemented HFD in which lipids came from palm oil. In this study, cholesterol over 1.25% was shown to promote steatosis, inflammation and fibrosis [60]. Addition of cholesterol in the diet was usually combined with cholate. The presence of cholic acid aids cholesterol, and fat absorption suppresses the conversion of cholesterol to bile acids and increases cholesterol levels (in particular, non-HDL-C). In female Wistar rats, animals fed by HFD+C diet (65% fat, 27% protein, 15% carbohydrates, 1% cholesterol, 0.25% cholate) for 16 weeks showed a NAS score equal to 6.2, with maximum grades for steatosis and inflammation, but only 0.7 for the ballooning criteria [61]. When cholate, but no cholesterol, was added to the diet (30% palm oil, 2% cholic acid and 68% of chow diet), low steatosis (grade 1–2), inflammation and ballooning in very few Sprague Dawley rats were observed after 9 weeks [60]. When the diet was complemented with cholesterol (1.25% or 2.5%), all histological NASH features were observed. Severity, incidence and homogeneity of these parameters were dependent upon the quantity of cholesterol contained in the diet, especially for ballooning and fibrosis. After 9 weeks, HFD+C showed no effect in body weight gain, blood serum insulin, glucose and triglycerides compared to HFD alone. After 18 weeks of the diet, body weight was lower than in the HFD control group [62]. Another group extended the study to 24 weeks with a similar diet (45.2% of total calories from fat, with 6.6% trans-fat as hydrogenated vegetable shortening, 2% cholesterol, total calorie density: 4600 kcal/kg) [63]. In their NASH model, NAS score was 6 and animals presented mild fibrosis. When Xu et al. extended the study to 48 weeks with a similar diet (30% of total calories from fat, mainly from lard oil, 2% cholesterol, total calorie density: 4800 kcal/kg), animals developed perisinusoidal fibrosis at 24 weeks and more severe fibrosis at 48 weeks, with fibrotic bridges in some rats [64]. Moreover, hyperglycemia and increased HOMA-IR were observed at 48 weeks. Nevertheless, the prolongation of the time of the diet did not improve NASH features and NAS score and was similar to the other studies. Similar observations were reported by Maciejewska et al. on the development of fibrosis at 20 weeks [65]. The atherogenic diet seems to be more efficient in rats than in mice, as 9 weeks of diet is sufficient to generate NASH and the development of fibrosis and cirrhosis in rats [66].

Taken together, these data showed that adding high levels of cholesterol (and cholate) in the diet seemed to accelerate and worsen fibrosis. However, the weakness of these HFD+C models was the difficulty in inducing ballooning, and longer HFD+C diet feeding did not appear to accentuate the diet-induced NASH, nor its progression into HCC, even though fibrosis occurred.

##### High-Fat and High-Sugar Diet

The consumption of sweetened beverages has increased over the last 30 years [67]. The two major sugar-sweetened beverages are sucrose (disaccharide fructose–glucose) and high-fructose corn syrup (monosaccharide of fructose and glucose). Increased consumption of sugar-sweetened beverages is closely associated with development of obesity, T2DM and dyslipidemia, such as NAFLD. In this particular case, the main component of the beverages is fructose. Fructose is one of the components that promote de novo lipogenesis pathway in the liver, leading to a higher lipid accumulation. Fructose can be metabolized into glucose, lactate and lipids independently of insulin action and can activate several key transcription factors such as SREBP-1c or carbohydrate-responsive element-binding protein (ChREBP) [68]. Fructose controls the activity of glucokinase; it is, therefore, a potent and acute regulator of liver glucose uptake and glycogen synthesis. By interfering with glucose metabolism, excessive fructose intake leads to postprandial hypertriglyceridemia, which increases visceral adipose deposition. Visceral adiposity contributes to hepatic triglyceride accumulation and insulin resistance by increasing the portal delivery of FFAs to the liver. Fructose leads to ATP depletion induced by rapid phosphorylation of fructose into fructose-1-phosphate (through fructokinase C) to finally produce uric acid responsible for undesirable metabolic effects, such as mitochondrial dysfunction and ER stress, which consequently results in oxidative stress [69,70]. Fructose promotes protein fructosylation and the formation of ROS in the liver [71]. Researchers used this complemented Western diet in order to better mimic the etiology of human NASH. A common strategy has been to add fructose, or high-fructose corn syrup, to the drinking water of rodents.

Wistar rats that were fed a high-fat and high-carbohydrate (HFHC, total calorie density: 4500 kcal/kg) diet composed of fat from soybean oil (49% of total calories), carbohydrates (35% of total calories), proteins (25% of total calories), 1.25% cholesterol, 0.5% cholate and 15% fructose in drinking water for 7 weeks showed glucose intolerance, higher plasma insulin levels and a higher HOMA-IR than the control group [72]. Rats developed obesity, hepatic steatosis and hypertrophy but very limited inflammation. A similar diet (total calorie density: 5420 kcal/kg, 60% fat from lard and corn oil, 10% sucrose in drinking water) was given to Sprague Dawley rats for 12 weeks [73]. The rats displayed obesity, hypertriglyceridemia, hypercholesterolemia, hyperinsulinemia and higher HOMA-IR. The NAS score of these animals was 6. In contrast to Henkel’s data, inflammatory infiltrate was observed in liver and hepatic TNF-α level was increased. In this study, treatment by genistein, an isoflavone phytoestrogen, showed reduced NAS histological features; a decrease in insulinemia and HOMA-IR, as well as cholesterol, in both serum and liver; a decrease in hepatic triglyceride contents; and a decrease in TNF-α and TLR4 protein and gene expression. Other researchers used a high-fat, high-glucose and high-fructose diet (HFHGFD, total calorie density: 5730 kcal/kg, 30% fat from butter, coconut oil, palm oil and beef tallow, 1% cholesterol and free access to a solution of 55% fructose and 45% glucose in drinking water) to feed Sprague Dawley rats for 8 weeks [74]. This diet induced obesity, hyperglycemia, hyperinsulinemia and higher HOMA IR, suggesting the presence of metabolic syndrome. Animals displayed liver injuries characterized by mild steatosis, mild ballooning and low inflammation. Heterogeneous results were observed in the histological scores, and NAS score did not exceed 5. This suggests that the sucrose administrated in drinking water allowed for the development of insulin resistance, unlike HF+C alone. Nevertheless, addition of these carbohydrates was not sufficient to worsen NASH after 8 weeks of diet. Similar results were observed by Tsuchiya et al. using a diet composed of 40% of total calories from fat (of which 94% were saturated fatty acids), 43% total calories from carbohydrates and 1% cholesterol during 12 weeks [75].

In mice, similar HFHS diets are described, i.e., the Amylin Liver NASH (AMLN) diet or American lifestyle-induced obesity syndrome (ALIOS) diet [76]. The AMLN diet (total calorie density: 4460 kcal/kg) is composed of 40% of total calories from fat (palm oil, soybean oil and lard) and 40% of total calories from carbohydrates, with 22% fructose (by weight) and 2% cholesterol. Mice fed with the AMLN diet for 20–30 weeks showed significant steatosis, mild inflammation and weak hepatocellular ballooning. The ALIOS diet contained 45% calories in the chow from fat, with 30% of the fat in the form of partially hydrogenated vegetable oil (28% saturated, 57% monounsaturated fatty acids (MUFAs), 13% polyunsaturated fatty acids (PUFAs) plus high-fructose corn syrup (55:45 *w*/*w* of fructose/glucose). After 16 weeks on the ALIOS diet, mice developed metabolic syndrome, hepatomegaly, hepatic dyslipidemia and macrovesicular steatosis. No evidence of inflammation or fibrosis was observed. In rat models, a similar diet seems to accentuate the presence of ballooning and inflammatory infiltrate at 8 weeks [74].

As described above, fructose was commonly used in combination with other stressors such as high fat and cholesterol. This strategy appears to promote steatosis and the progression to NASH. Although closer to the etiopathogenesis of human NAFLD despite increased fructose consumption being associated with fibrosis severity in patients with NAFLD [77], these HFHS models failed to initiate liver fibrosis.

## 3. Other Factors Affecting Diet-Induced NAFLD/NASH Model Phenotype

### 3.1. Effects of Food Intake Methods

Control of food and calorie intake by rats is not easy to standardize in normal housing conditions. To solve this problem, Zou et al. [27] proposed feeding the animals through gavages of a liquid diet. The diet, called high-fat emulsion (HFE), is composed of 77% of total calories from fat derived from corn oil, 10% cholesterol and 2% cholate with a total energy of 4342 kcal/L and is administered orally. Sprague Dawley rats were fed this diet for 6 weeks, with free access to chow diet, water and a saccharose solution (18% *w*/*v*). They developed obesity, hyperinsulinemia, hyperglycemia and hypertriglyceridemia, associated with NASH. In 2017, Guo et al. performed a similar study with an HFE composed of 20% lard, 10% cholesterol, 2% sodium cholate, 0.5% propylthiouracil and 30% fructose [78]. However, in contrast to the experiment by Zou et al., the rats did not develop NASH in this case. This discrepancy might be explained by the composition of the HFE, because fat originated from lard instead of corn oil and carbohydrates were mainly fructose instead of saccharose. Recently, Karimi-Sales et al. [79] provided HFE to male Wistar rats for 6 weeks. The diet induced hepatic steatosis, inflammation and ballooning, leading to NASH (NAS score = 6.5). The HFE diet appeared to be useful in controlling the food calorie intake and counteracted the rats’ adaptation to calorie consumption that may be a limitation for the development of NASH animal models.

Another way to counteract the rats’ adaptation to calorie consumption is to provide animals with a diet that mimics the “cafeteria diet” (CAF) in a diversity of forms, tastes and textures. Sampey et al. [80] fed male Wistar rats with standard chow complemented with cookies, cereals, cheese, processed meats and crackers ad libitum and fed the control group with HFD (45% of fat from lard). After 15 weeks, the CAF-fed animals displayed dramatic obesity, hyperinsulinemia, hyperglycemia and glucose intolerance and remarkable inflammation in white fat, brown fat and the liver, compared to the HFD rats. For a rodent, the texture of this CAF food might be more appealing than the soft texture of regular rodent HFD.

### 3.2. Effect of Housing Environmental Factors

Housing rodents at thermoneutrality reverses multiple physiological effects of cold stress, including reduction in heart rate and overall energy expenditure. Compared to standard housing conditions (19–22 °C), mice housed under thermoneutral conditions (30–32 °C) displayed a proinflammatory immune response but worsened HFD-induced NASH progression. Additionally, mice displayed increased intestinal permeability and alterations in gut microbiome, features mimicking the human NAFLD [81]. In Sprague Dawley rats, the housing temperature had a marked influence on the effect of dietary (HFHS)-induced rise in fat deposition without affecting body weight [82].

The circadian clock disruption is clearly involved in human diseases such as metabolic syndrome. Indeed, shift workers more frequently develop health problems such as metabolic syndrome [83] and abnormal blood lipids [84]. It has been previously shown that the circadian rhythm drives oscillations in hepatic triglyceride levels, inflammation, oxidative stress, mitochondrial dysfunction and hepatic insulin resistance [85,86]. Moreover, it has recently been suggested that chronic disruption of the circadian rhythm may spontaneously induce the progression from NAFLD to NASH, fibrosis and HCC [87,88]. Yamajuku et al. showed that suppression of a regular feeding rhythm in Wistar rats increased the secretion rate of VLDL cholesterol from the liver and decreased the excretion of fecal bile acids [89]. The same team established a delayed first active-phase meal (DFAM) protocol as a breakfast-skipping model. Wistar rats were fed an HFD at zeitgeber time (ZT) 12–24 in a control group and ZT 16–4 in the DFAM group. The DFAM group showed obesity and perirenal adipose tissue weight gain, without change in total food intake between the groups [90]. These results suggest that the delayed circadian rhythm of clock genes and lipid metabolism leads to increased body and adipose tissue weights.

### 3.3. Sex-Related Differences

The prevalence and severity of NAFLD and NASH are higher in men than in women during the reproductive age; the same is observed for the incidence of HCC. However, after menopause, NAFLD occurs at a higher rate in women, suggesting the protective effect of estrogen [91]. In general, rodent models of NAFLD and NASH recapitulate these sex differences. Males appear more susceptible to the development of NAFLD by HFD feeding, with more severe steatosis and steatohepatitis [92,93] and higher susceptibility to metabolic disorders in male mice than females [94]. Similarly, MCDD-fed male rats developed greater steatosis, liver cell injury and inflammation than female rats [31].

Incorporating sex differences in study design and analytic strategy represents methodological challenges. Including both males and females increases the number of animals and the cost of the experiments. However, to recapitulate human NAFLD/NASH, sex-inclusive research is necessary.

### 3.4. The Role of Gut Microbiome

Recent years brought enhancing understanding that the interaction between the physiology of the gut and the liver, called gut–liver axis, plays important role in the pathogenesis of NAFLD and NASH [95]

The gut microbiota composition and function are shaped by a variety of host and environmental factors, including diet. It was demonstrated that rats fed HFD have different microbiota composition compared to those fed control diet, and the modifications in gut microbiome correlate with metabolic parameters [96]. García-Lezana et al. showed that intestinal microbiota transplantation from rats fed a control diet restored a healthy intestinal microbiota and normalized portal hypertension in rats fed HFHGFD [74]. Importantly, the composition of gut microbiota can be altered by bile acids, inhibitors against bile acid absorption and farnesoid x receptor (FXR) agonists. Indeed, it was demonstrated that cholic acid administration modifies the composition of the gut microbiota in rats [97] and that the treatment with obeticholic acid, an FXR agonist, leads to improved insulin resistance and decreased liver steatosis in NAFLD rat model [98]. Today, diet-induced NAFLD and NASH models are widely used in research focused on the causal role of gut microbiota in NAFLD/NASH development and the possible therapeutic interventions.

## 4. Conclusions

**Figure 3:**
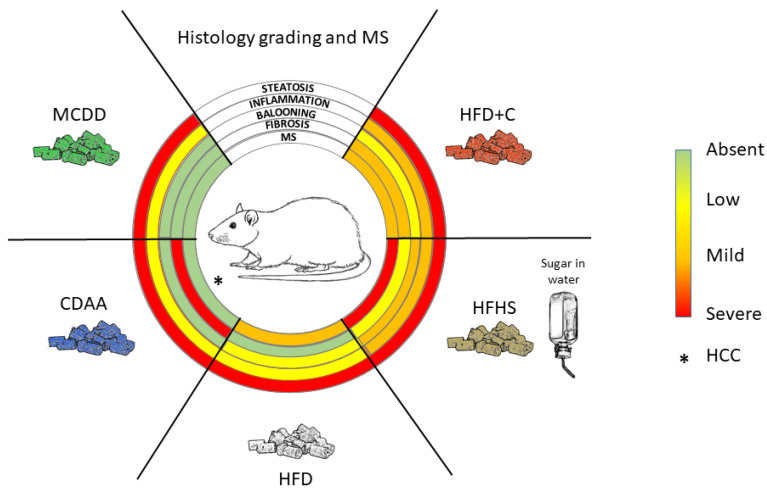
Key nonalcoholic fatty liver disease (NAFLD)/nonalcoholic steatohepatitis (NASH) histological features observed in different diet-induced rat models. HFD+C: high-fat diet plus cholesterol; HFHS: high-fat and high-sugar; HFD: high-fat diet (variability of phenotype exists due to different origins of fat in the diet composition; only average phenotype is represented); CDAA: choline-deficient L-amino acid defined; MCDD: methionine- and choline-deficient diet; MS: metabolic syndrome (blood pressure rarely assessed). * HCC: hepatocellular carcinoma has been observed in this model.

As reviewed in this paper and summarized in Figure 3, a large number of diet-induced NAFLD/NASH models have been developed in rats. However, they closely mimic mouse models, and no original approaches have been proposed so far. Interestingly, with the exception of the MCDD model, dietary models were usually more efficient in triggering liver and systemic pathologies related to NAFLD and NASH in rats than in mice. However, one should keep in mind that these models are difficult to compare and replicate because of divergences in protocol length, diet ingredient origins and composition (which bear seasonal variations), genetic backgrounds of the animals, age of the animals and other uncontrolled parameters. The effects of external factors such as temperature or the circadian clock on NAFLD and NASH development in rats have been poorly described. More experiments should be performed to consolidate the influence of these factors for preclinical models based on rats. Nevertheless, the majority of these nutritional models do not completely reflect the human diet, rarely developed advanced fibrosis and did not lead to HCC, the end-point progression of NASH.

Limiting factors in developing transposable models are the time frame required in humans to establish a certain liver disease and the different metabolic rates affecting liver homeostasis. In this context, conciliating the highest relevance of diet-induced NAFLD models with the time constraints of drug testing in preclinical models might represent an unreachable goal, and the low percentage of patients that progress to NASH worsens the problem. Nevertheless, current bad dietary habits and increasing incidence of metabolic syndrome worldwide urge us to improve our in vivo approaches and continue our efforts to propose highly transposable animal models. In regard to the current published data, rat models might be helpful in such a quest.

Each model has its strengths and shortcomings. To answer a specific research question or to test a specific therapeutic compound, one should select the most appropriate model corresponding to the stage of development of the liver disease being targeted.
